# Risk stratification of spontaneous bacterial peritonitis recurrence: integrating acute kidney injury, biomarkers, composite scores, and machine learning models

**DOI:** 10.1186/s13099-025-00774-5

**Published:** 2025-12-07

**Authors:** Nasser Mousa, Alaa Elmetwalli, Mostafa Abdelsalam, Mohamed Wahba, Mohamed Selim, Dina Nour, Eman Abdelkader, Ahmed El-Eraky, Amany Hasson, Ahmed E Taha, Eman Mousa, Adel El-Assmy, Ali El-Assmy, Sherif Shiha, Muhammad Diasty, Mohammed Abdelaziz, Shereen A. Mourad, Nader Elmalky, Marwa Mansour

**Affiliations:** 1 Tropical Medicine Department, Mansoura University, Mansoura, Egypt; 2 Egyptian Liver Research Institute and Hospital (ELRIAH), Mansoura, Egypt; 3 Prince Fahad bin Sultan Research Chair for Biomedical Research, University of Tabuk, Tabuk, Saudi Arabia; 4 Internal Medicine, Nephrology and Dialysis Unit, Mansoura University, Mansoura, Egypt; 5 Alameen General Hospital, Taif, Saudi Arabia; 6 internal Medicine Department, Mansoura University, Mansoura, Egypt; 7 Internal Medicine Department, Dr. Soliman Fakeeh Hospital, Riyadh, Saudi Arabia; 8 Microbiology and Immunology unit, Department of Pathology, College of Medicine, Jouf University, Sakaka city, Saudi Arabia; 9 Medical Microbiology and Immunology Department, Faculty of Medicine, Mansoura University, Mansoura, Egypt; 10 Faculty of Dentistry, Mansoura University, Mansoura, Egypt; 11 Intern doctor, Faculty of Medicine, Mansoura University, Mansoura, Egypt; 12 Department of Clinical Pathology, Faculty of Medicine, Mansoura University, Mansoura, Egypt; 13 Internal Medicine Department, Faculty of Medicine, Jouf University, Sakaka city, Saudi Arabia

**Keywords:** Recurrent spontaneous bacterial peritonitis, Acute kidney injury, Inflammatory markers, Machine learning, Composite scoring, Predictive modeling

## Abstract

**Background and aim:**

Recurrent spontaneous bacterial peritonitis (SBP) is a major concern for cirrhotic patients with ascites. This study seeks to identify predictors of recurrent SBP using clinical factors, inflammatory markers, and machine learning models.

**Patients and methods:**

The study involved 347 patients with cirrhotic ascites and SBP. Receiver Operating Characteristic (ROC) curve analysis assessed the predictive ability of biomarkers. A composite score was created to evaluate the risk stratification model. Different machine learning models were compared for predictive accuracy.

**Results:**

Eighty-three patients (23.9%) experienced recurrent SBP. Independent predictors of recurrence in multivariable analysis included acute kidney injury (AKI), elevated C-reactive protein (CRP) levels, higher serum bilirubin levels, a higher model for end-stage liver disease (MELD) score, proton-pump inhibitor (PPI) use, and lack of β-blocker use. A composite 10-point score (including AKI, CRP > 50 mg/L, low albumin levels < 2.5 g/dL, ascitic protein < 1.0 g/dL, albumin/ascitic ratio < 2.5 [2 points], MELD ≥ 15, diabetes, multidrug-resistant organism [MDRO] infection, and non-use of β-blockers) stratified the risk of recurrence into low (0–3: 15%), moderate (4–6: 45%), and high (7–10: 80%) categories. Machine learning models outperformed supervised machine logistic regression in predicting recurrence. Logistic regression achieved 70% accuracy, 65% sensitivity, and 68% specificity. The decision tree model improved accuracy to 75%, sensitivity to 72%, and specificity to 71%. The random forest model showed the best performance with 78% accuracy, 77% sensitivity, and 76% specificity.

**Conclusion:**

A composite score, combined with machine-learning models like random forest, enhances risk assessment for SBP recurrence. Clinical predictors such as AKI, CRP, bilirubin, MELD, PPI use, and β-blockers non-use help in targeted prevention.

## Introduction

Liver cirrhosis is a significant global health concern that leads to complications such as ascites, infections like spontaneous bacterial peritonitis, gastrointestinal bleeding, portal vein thrombosis, and hepatocellular carcinoma. Early detection and treatment of these conditions are essential for better patient outcomes and reducing mortality rates in advanced liver disease [1–4].

Spontaneous bacterial peritonitis (SBP) is a serious complication of cirrhosis and ascites. The prevalence of SBP is 1.5–3% in outpatients and can increase to up to 37% in hospitalized patients [5–8]. The SBP is a significant predictor of mortality, with rates ranging from 20% to over 50% at 6 and 12 months, respectively [9]. Despite prompt antibiotic treatment, SBP carries significant risks, including high rates of recurrence, which can lead to acute kidney injury-hepatorenal syndrome, repeated hospitalizations, multi-organ failure, and increased mortality. The recurrence rate of SBP within one year of the initial presentation is reported to be between 10% and 30%, and it is associated with a very high mortality rate emphasizing the urgent need for effective strategies to predict and prevent these recurrences [10–12].

Finding reliable predictive markers for SBP recurrence is challenging due to the lack of a single sufficient biomarker. Therefore, there is a need for a non-invasive and highly accurate tool to diagnose and predict SBP recurrence. Current efforts to identify predictive markers have centered on parameters like serum albumin levels, ascitic fluid protein, inflammatory markers, and antibiotic prophylaxis [13, 14]. However, no single biomarker has reliably differentiated between high and low-risk patients. Clinical and laboratory risk factors for SBP development, such as a history of SBP [15], variceal hemorrhage [16], proton pump inhibitor use [17], C-reactive protein [18], and serum creatinine levels [19], are known, but data are inconsistent. Non-invasive methods for predicting SBP show promise and are becoming increasingly important. Simple composite scoring systems involving multiple easily collected parameters at hospital admission are more effective in measuring disease progression compared to rely on individual biomarkers [20]. Supervised machine learning models like random forest, decision tree, and logistic regression offer a solid framework for identifying crucial digital features that indicate disease progression. These techniques facilitate the development of composite metrics that effectively track disease advancement [21]. When it comes to recurrent SBP, integrating biomarkers, composite scoring systems, and machine learning models holds promise for recurrence prediction. The underexplored aspect of predicting recurrent SBP through this integrated method underscores the need for further research. More exploration is necessary to determine how advanced analytical tools can bolster risk stratification and enhance patient outcomes in this clinical context.

This study aims to develop a reliable tool for predicting recurrent SBP in clinical practice by evaluating clinical variables, inflammatory markers, and supervised machine learning models.

## Patients and methods

This cohort study included 347 cirrhosis patients with ascites, aged 18 years and older, diagnosed with spontaneous bacterial peritonitis (SBP). The study was conducted at the Tropical and Internal Medicine departments, Mansoura University, Mansoura, Egypt, in collaboration with the Egyptian Liver Hospital (ELRIAH), Sherbin, Mansoura, Egypt, from February 2024 to March 2025. Patients experiencing their first episode of SBP received treatment according to international guidelines [22]. Following improvement in ascitic fluid count and clinical condition, they were discharged and monitored for 6 months to assess recurrence rates and predictive markers. All discharged patients were prescribed prophylactic antibiotics. Baseline laboratory data, including serum albumin and ascitic fluid protein levels, were available for all included patients. Approval was obtained from the Institutional Review Board (IRB) at ELH with the approved number #CT-2024-005, and all procedures adhered to privacy regulations.

### Exclusion criteria

Patients with non-cirrhotic ascites due to conditions such as heart or renal failure, advanced malignancies (including hepatocellular carcinoma), sepsis, secondary bacterial peritonitis, and those with unrelated treated infections (skin and lung infections), immunocompromised individuals, and patients with acute variceal hemorrhage were excluded. Patients who died during the follow-up period or had missing information on readmission were also excluded.

### Data collection

Baseline demographic, laboratory, and clinical data were collected, including age, sex, comorbidities, and Model for End-Stage Liver Disease (MELD) score. Patients were monitored for a six-month period to detect recurrent SBP, which was defined as the occurrence of a second episode following the initial infection.

### Diagnosis of spontaneous bacterial peritonitis

SBP was diagnosed based on international guidelines, with a polymorphonuclear (PMN) cell count in the ascitic fluid ≥ 250/m^3^ and positive ascitic fluid culture (culture-positive SBP) or with PMN ≥ 250/m^3^ and a negative ascitic fluid culture (culture-negative neutrocytic ascites) in the absence of other causes of peritonitis and hemorrhagic ascites [22, 23]. Regarding microbiological examination, inoculated blood culture bottles were incubated for three successive days at 37 °C with daily subculture on blood, MacConkey, and chocolate agars. Antimicrobial susceptibility testing and bacterial identification were carried out using standard procedures.

### Diagnosis of acute kidney injury (AKI)

In this study, the international Club of Ascites (ICA) and the Acute Disease Quality Initiative (ADQI) in patients with cirrhosis and ascites using the Kidney Disease Improving Global Outcomes (KDIGO) criteria was followed. AKI in this population is characterized by: (1) cirrhosis with ascites; (2) a rise in serum creatinine (SCr) of ≥ 0.3 mg/dl within 48 h or ≥ 50% from baseline within the prior 7 days, along with urine output (UO) ≤ 0.5 ml/kg for ≥ 6 h; (3) lack of improvement in SCr and/or UO within 24 h after adequate volume resuscitation (if clinically indicated); and (4) no strong evidence supporting an alternative cause for AKI. Diagnosis of AKI was based on ICA-ADQI criteria within 48 h to 7 days, depending on changes in serum creatinine levels [24]. The baseline was clarified as admission values during the first SBP episode.

### Biochemical analysis methods

Ascitic fluid and blood samples were collected from each patient during their initial SBP episode following standard sterile procedures. Serum and ascitic fluid albumin levels were measured using an automated biochemical analyzer (StatLab2, Spectrum Diagnostics, Cairo, Egypt; catalog no. ALB-250) with spectrophotometric methods based on bromocresol green dye-binding, a widely accepted method for accurate quantification. Total protein levels in the ascitic fluid were also measured using the biuret method, which provides precise protein concentration by forming a colored complex between peptide bonds and copper ions under alkaline conditions. These assays were calibrated against standard references to ensure reliability across measurements. The albumin-to-ascitic fluid protein ratio was calculated for each patient by dividing the serum albumin concentration by the ascitic fluid protein concentration, a value previously suggested to reflect the peritoneum’s immune-nutritional status and barrier function [25]. Additionally, routine liver function tests, such as total bilirubin, alanine aminotransferase (ALT), aspartate aminotransferase (AST), and serum creatinine, were included in the biochemical analyses to assess liver disease severity and renal function, both of which can influence SBP risk and recurrence. This biochemical analysis aimed to establish baseline values and determine whether these biomarkers, individually and in ratio form, could predict recurrent SBP.

### Predictive parameters and scoring model development

Key predictors of recurrence, such as acute kidney injury (AKI), C-reactive protein (CRP), serum albumin, ascitic fluid protein, and the albumin/ascitic fluid protein ratio, were assessed at baseline. A composite score was then created, taking into account additional risk factors like diabetes and multidrug-resistant organisms (MDROs). This scoring system was designed to enhance prediction accuracy beyond individual markers.

### Composite score derivation

To create a clinically interpretable bedside risk tool, we assigned weighted points to predictors based on their relative contribution and clinical relevance to spontaneous peritonitis. Each factor received 1 point, except for the albumin/ascitic protein ratio < 2.5, which was given 2 points due to its strong association with ascites formation and fluid defense mechanisms against infection. Hypoalbuminemia from decompensated liver disease leads to a decrease in oncotic pressure, further promoting ascites development [26]. Additionally, ascitic proteins like Complement 3 (C3) play a crucial role in local defense against bacterial infection particularly spontaneous bacterial peritonitis and provide direct anti-bacterial defense within the ascitic fluid in cirrhotic patients [27]. MELD ≥ 15 was weighted 1 point to maintain the clinical focus on protein status while retaining overall prognostic balance. The total score ranged from 0 to 10. Risk groups were defined as low (0–3), moderate (4–6), and high (7–10) based on recurrence incidence observed in the cohort.

### Data set and preprocessing

We analyzed 347 patients (observations) with 10 baseline features (clinical/biochemical/inflammatory). The missing data were < 5% across all variables and thus imputation was appropriate. We used Multiple Imputation by Chained Equations (MICE) because it preserves the variability and correlation structure of the data, thereby reducing potential bias compared to complete-case analysis. To ensure robustness, we conducted sensitivity analyses by comparing results from the imputed dataset with those from a complete-case analysis. These analyses showed no meaningful differences in effect estimates or predictive model performance, supporting the validity of our approach. Because recurrence was less frequent, we addressed class imbalance using SMOTE on the training folds only. Relevant text has been added to the Methods section.

### Modeling evaluation

We evaluated:

- **Logistic regression (baseline supervised machine learning model)**. L2-penalized logistic regression (C = 1.0) with AIC-guided stepwise selection on the training set.
- **Decision tree.** Gini criterion; max_depth = 5; min_samples_split = 2.
- **Random forest.** n_estimators = 500; max_features = sqrt(p); random_state = 42.

### Train/validation/testing

Data were split 70/30 into training/test sets (stratified by outcome). Hyperparameters were tuned by 5-fold cross-validation within the training set; the held-out 30% test set was used for final performance reporting (accuracy, sensitivity, specificity, AUC).

### Comparative statistics

For paired ROC AUCs (same patients), we compared models using DeLong’s test. For paired accuracy/sensitivity/specificity, we used McNemar’s test on matched predictions—two-sided α = 0.05 with Bonferroni correction for multiple pairwise comparisons. Survival/time-to-event models were not used because recurrence was assessed once at 6 months (binary endpoint). Analyses were run in R 4.3.2 (caret, pROC, glmnet, random forest, e1071) and Python 3.10 (scikit-learn 0.24.2, pandas 1.2.3, matplotlib 3.4.2).

### Statistical analysis

#### Descriptive statistics

Baseline characteristics were summarized using means and standard deviations (SD) for normally distributed continuous variables, medians with interquartile ranges (IQR) for non-normally distributed variables, and frequencies with percentages for categorical variables. Group comparisons between recurrent and non-recurrent SBP were performed using the Student’s t-test for normally distributed continuous variables and the Mann–Whitney U test for skewed distributions. Chi-square tests (or Fisher’s exact test when appropriate) were used for categorical variables.

#### Model performance metrics

Predictive performance of logistic regression (baseline supervised model), decision tree, and random forest models was assessed using accuracy, sensitivity, specificity, and the area under the receiver operating characteristic curve (AUC). AUC values quantified model discrimination between recurrent and non-recurrent SBP. All metrics were calculated on the independent 30% test set after hyperparameter tuning via cross-validation within the training set.

### Statistical tests for model comparison

DeLong’s test was used to compare paired ROC AUCs between models, while McNemar’s test was applied for paired proportions (accuracy, sensitivity, specificity) on matched predictions. Statistical significance was set at *p* ≤ 0.05, with Bonferroni correction applied for multiple pairwise comparisons to control for type I error. A classification decision tree was implemented using the CART (Classification and Regression Tree) algorithm. The Gini impurity criterion was applied to evaluate splits at each node. To reduce overfitting, the maximum depth of the tree was restricted to 5 levels, and the minimum number of samples required to split an internal node was set to 2. These parameters were chosen to balance interpretability with predictive performance.

The outcome of interest was recurrence of SBP within a fixed 6-month follow-up period after the initial episode, defined as a binary variable (recurrence: yes/no). Because all predictors (clinical, biochemical, and inflammatory markers) were measured at baseline and recurrence was assessed once at the end of follow-up, this study design did not generate repeated longitudinal measurements. Therefore, time-series or survival analysis models were not applicable. Instead, classification models were used to estimate recurrence risk within the defined follow-up window.

## Results

**Fig. 1:**
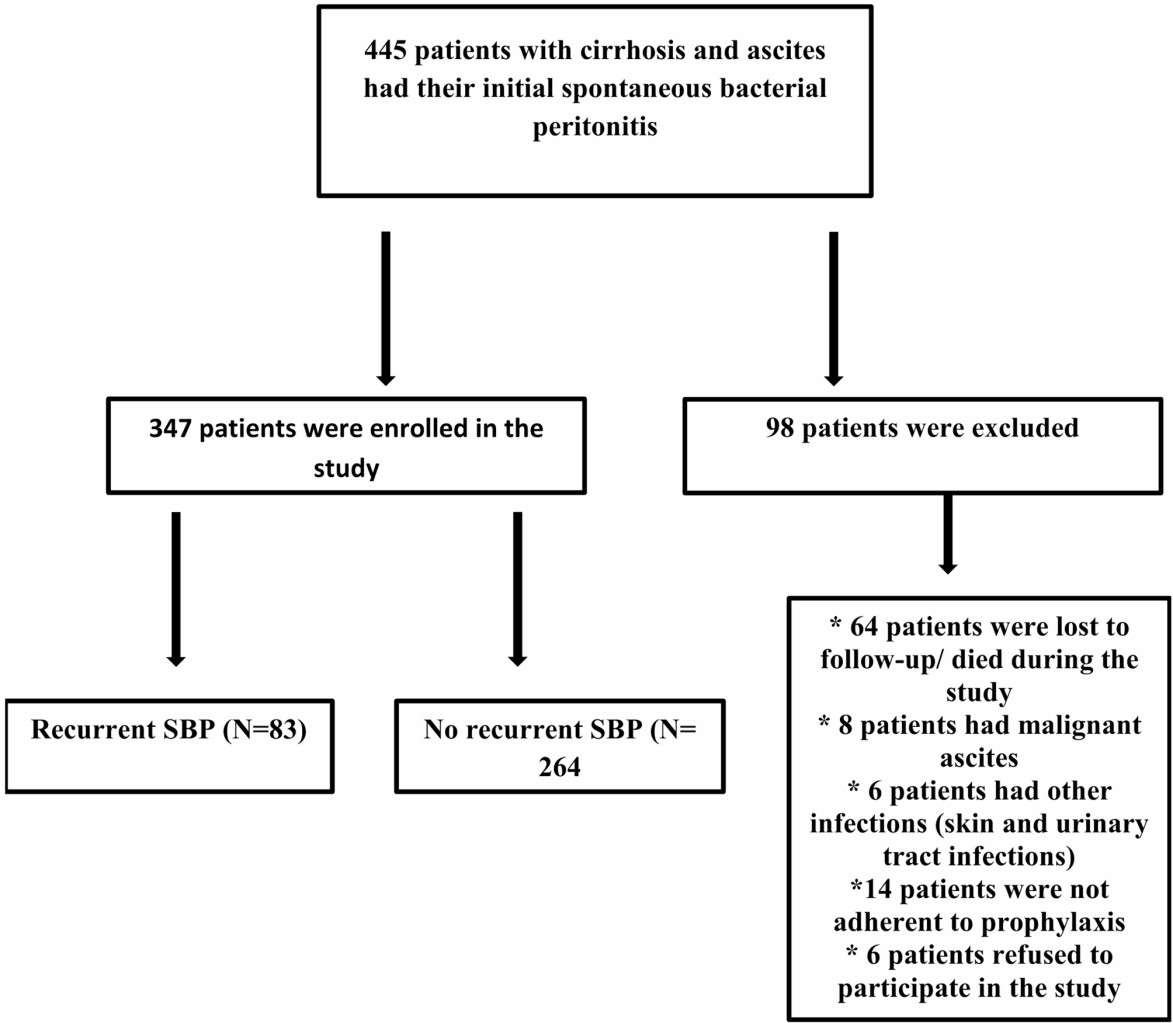
Study flow diagram. A total of 445 patients with cirrhosis and ascites who experienced an initial episode of spontaneous bacterial peritonitis (SBP) were screened. After applying exclusion criteria (*n* = 98; including loss to follow-up or death, malignant ascites, other infections, non-adherence to prophylaxis, or refusal to participate), 347 patients were enrolled. Of these, 83 (23.9%) developed recurrent SBP and 264 (76.1%) did not experience recurrence

The study initially involved 445 patients with cirrhotic ascites and spontaneous bacterial peritonitis (SBP). After excluding those who did not meet the inclusion criteria, 347 patients were included in the final analysis. Among them, 83 patients (23.91%) experienced recurrent SBP, while 264 patients (76.08%) had non-recurrent SBP. Ninety-eight patients were excluded from the study, 64 patients were lost to follow-up/died, 8 patients had malignant ascites, 6 patients had other infections (skin and urinary tract infections), 14 patients were not adherent to prophylaxis, and 6 patients refused to participate (Fig. 1). Among the patients with recurrent SBP, *Escherichia coli*, a gram-negative bacterium, was the most commonly isolated organism in 53 patients (63.85%). *Streptococcus pneumoniae* was found in 9 patients (10.84%), *Staphylococcus species* in 8 patients (9.63%), *Pseudomonas species* in 5 patients (6.02%), Group D *Streptococcus* in 5 patients (6.02%), and *Listeria monocytogenes* in 3 patients (3.61%).

### Data interpretations of the studied cohort

The analysis of clinical and laboratory parameter differences between non-recurrent and recurrent groups of patients with SBP is presented in Table 1. There were no significant differences in age, ascites polymorphonuclear leukocytes (PMN), ascites lymphocytes, blood white blood cell (WBC), blood PMN, blood lymphocytes, and hemoglobin levels (*p* > 0.05). However, inflammatory markers such as CRP and ascites WBC counts were significantly higher in the recurrent group, indicating a more pronounced inflammatory response in recurrent cases. The recurrent group also had significantly lower serum albumin levels and platelet counts, as well as higher rates of acute kidney injury (AKI), serum creatinine, serum bilirubin, prothrombin time, and MELD score compared to the non-recurrent group. The group with recurrent SBP had a significantly lower rate of β-blockers usage and a higher rate of proton pump inhibitor usage, diabetes mellitus, fever, and multidrug-resistant organisms compared to the non-recurrent SBP group.

**Table 1:** Characteristics of the recurrence and non-recurrence groups of spontaneous bacterial peritonitis

Variable	Non-Recurrent (*N* = 264) Mean (± SD)	Recurrent (*N* = 83) Mean (± SD)	*P*-Value
**Age (years)**	55.70 (± 8.60)	55.60 (± 7.70)	0.92
**CRP (mg/L)**	42.60 (± 3.50)	51.10 (± 3.03)	0.04
**Ascitic Fluid Protein (g/dL)**	1.10 (± 0.30)	0.8 (± 0.20)	0.03
**Ascites WBC**	4.09 (± 0.71)	4.64 (± 0.50)	0.03
**Ascites PMN (%)**	69.00 (± 11.10)	66.8 (± 8.10)	0.21
**Ascites Lymph (%)**	31.70 (± 12.50)	33.7 (± 8.40)	0.18
**Blood WBC**	10.04 (± 0.80)	10.22 (± 0.50)	0.07
**Blood PMN (%)**	68.60(± 13.90)	71.70 (± 12.40)	0.15
**Blood Lymph (%)**	18.20 (± 10.50)	16.2 0(± 8.50)	0.19
**Hemoglobin (g/dL)**	10.30 (± 2.10)	10.00 (± 1.80)	0.22
**Platelet (cells/mL)**	97.54 (± 70)	93.50 (± 50)	0.05
**Serum Albumin (g/dL)**	3.10 (± 0.70)	2.00 (± 0.50)	< 0.001
**Serum Bilirubin (mg/dL)**	2.40 (± 0.80)	3.10 (± 1.10)	0.02
**Serum Creatinine (mg/dL)**	1.05 (± 0.20)	1.40 (± 0.30)	0.01
**AKI (%)**	26 (9.80%)	20 (24.10%)	0.03
**Prothrombin Time (sec)**	16.70 (± 2.90)	19.20 (± 3.30)	0.02
**MELD Score (Mean ± SD)**	14.20 (± 3.10)	16.80 (± 3.60)	0.02
**Albumin/Ascitic Fluid Protein Ratio**	2.80 (± 0.90)	2.50 (± 0.70)	0.005
**Diabetes Mellitus (N/%)**	53/20	30/36	0.03
**Multi Drug-Resistant bacteria (N/%)**	34/13	19/23	0.04
**β-blockers usage (N/%)**	186/70	40/48	0.03
**Proton pump inhibitors usage (N/%)**	150/57	56/68	0.04
**Presence of Fever (N/%)**	129/49	63/76	0.02

Values are expressed as mean ± standard deviation (SD) for continuous variables and percentages for categorical variables Univariate comparisons (t-test/Mann-Whitney or χ²)

### Significant predictors of recurrence

Table 2 summarizes the predictors associated with SBP recurrence. AKI was a strong independent predictor (*p* = 0.03, OR 3.1, 95% CI: 1.3–7.4), indicating that patients with AKI had over threefold higher odds of recurrence compared to those without AKI. CRP was also independently associated with recurrence (*p* = 0.04, OR 1.05, 95% CI: 1.01–1.10), with each unit increase in CRP corresponding to a 5% increase in recurrence risk. Serum bilirubin was another significant factor (*p* = 0.02, OR 1.4, 95% CI: 1.1–2.3), showing that for every 1 mg/dL rise in bilirubin, recurrence odds increased by approximately 40%.

**Table 2:** Predictors of SBP recurrence

Predictor Variable	Non-Recurrent Mean (± SD)	Recurrent Mean (± SD)	Odds Ratio (95% CI)
**AKI (%)**	9.80%	24.10%	3.1 (1.30–7.40)
**CRP (mg/L)**	42.60 (± 35.8)	51.10 (± 3.30)	1.05 (1.01–1.10)
**Serum Albumin (g/dL)**	3.10 (± 0.7)	2.00 (± 0.50)	1.1 (0.93–1.26)
**Albumin/Ascitic Fluid Protein Ratio**	2.80. (± 0.9)	2.50 (± 0.70)	0.80 (0.51–1.08)
**Serum Bilirubin (mg/dL)**	2.40 (± 0.8)	3.10 (± 1.10)	1.4 (1.10–2.30)
**β-blockers usage (N/%)**	186/70	40/48	2.5 (1.20–5.30)
**Proton pump inhibitors usage (N/%)**	150/57	56/68	1.6 (1.0–2.80)
**MELD Score (Mean ± SD)**	14.20 (± 3.1)	16.80 (± 3.60)	2.9 (1.40–6.10)

Odds Ratios (OR) with 95% Confidence Intervals (CI) were calculated using binary logistic regression analysis. P-values were obtained using the Chi-square test for categorical variables and Student’s t-test for continuous variables

Medication use also played a role. Non-use of β-blockers was significantly more common among recurrent cases (*p* = 0.03, OR 2.5, 95% CI: 1.2–5.3), suggesting a protective effect of β- β-blockers. Conversely, PPI use was higher in recurrent cases (*p* = 0.04, OR 1.6, 95% CI: 1.0–2.8), identifying it as a potential risk factor. MELD score was independently associated with recurrence (*p* = 0.02, OR 2.9, 95% CI: 1.4–6.1), with each additional point almost tripling the odds of recurrence. Other variables showed associations in univariate analyses but did not remain independent after adjustment. Lower serum albumin levels were more common in recurrent cases (*p* < 0.001), but the confidence interval for its odds ratio (OR 1.1, 95% CI: 0.93–1.26) crossed 1.0, indicating it was not an independent predictor. Similarly, a lower albumin/ascitic fluid protein ratio appeared protective (*p* = 0.005, OR 0.80, 95% CI: 0.51–1.08), but the wide CI that included 1.0 suggests it was not independently predictive after adjustment.

### Composite scoring system for SBP recurrence prediction

A composite scoring system was developed to predict the recurrence of SBP (Table 3). Points were assigned based on predefined clinical thresholds for nine parameters. The presence of AKI, CRP > 50 mg/L, serum albumin < 2.5 g/dL, and ascitic fluid protein < 1.0 g/dL each contributed 1 point. The albumin/ascitic protein ratio < 2.5 was assigned 2 points, reflecting its strong clinical relevance in the pathophysiology of spontaneous peritonitis. MELD score ≥ 15, diabetes mellitus, multidrug-resistant organisms (MDRO), and non-use of β-blockers each contributed 1 point. This scoring system produces a maximum of 10 points, allowing patients to be stratified into distinct recurrence risk categories: low risk (0–3 points, ≈ 15% recurrence), moderate risk (4–6 points, ≈ 45% recurrence), and high risk (7–10 points, ≈ 80% recurrence). By integrating inflammatory, biochemical, and clinical predictors into a single tool, the composite score provides a practical and comprehensive framework for identifying patients at the highest risk of SBP recurrence.

**Table 3:** Score for predicting SBP recurrence

Parameter	Points Assigned	Thresholds
**AKI**	1	Yes
**C-Reactive Protein (CRP)**	1	> 50 mg/L
**Serum Albumin Level**	1	< 2.5 g/dL
**Ascitic Fluid Protein Level**	1	< 1.0 g/dL
**Albumin/Ascitic Fluid Protein Ratio**	2	< 2.5
**MELD Score**	1	≥ 15
**Presence of Diabetes Mellitus**	1	Yes
**Presence of Multi-Drug-Resistant bacteria**	1	Yes
**Non-use of β-blockers**	1	Yes

Points were assigned to predictors based on predefined clinical thresholds and their relative contribution to recurrence risk. The scoring system includes: Acute kidney injury (AKI), CRP > 50 mg/L, serum albumin < 2.5 g/dL, ascitic fluid protein < 1.0 g/dL, MELD ≥ 15, diabetes mellitus, multidrug-resistant organisms (MDRO), and non-use of β-blockers (each = 1 point). Albumin/ascitic protein ratio < 2.5 (assigned 2 points, reflecting its strong biological relevance to ascitic fluid defense mechanisms). The maximum composite score is 10 points.

### Recurrence risk stratification based on composite scores

Table 4 classifies patients into low, moderate, and high-risk categories based on their composite score. Patients with scores of 0–3 are considered low risk, with a 15% recurrence rate. Those scoring 3–6 are categorized as moderate risk, with a 45% recurrence rate. Patients with scores between 6 and 10 are classified as high risk, with an 80% recurrence rate. This stratification method demonstrates the predictive value of composite scoring, where higher scores are associated with a higher likelihood of recurrence, facilitating patient care and monitoring.

**Table 4:** Risk stratification based on composite score

Score Range	Risk Level	% Recurrent Cases
**0–3**	Low	15%
**4–6**	Moderate	45%
**7–10**	High	80%

Patients are stratified into three risk categories:

Low risk: 0–3 points (≈ 15% recurrence)

Moderate risk: 4–6 points (≈ 45% recurrence)

High risk: 7–10 points (≈ 80% recurrence)

### ROC curve analysis of predictive discriminators: value of biomarkers

**Fig. 2:**
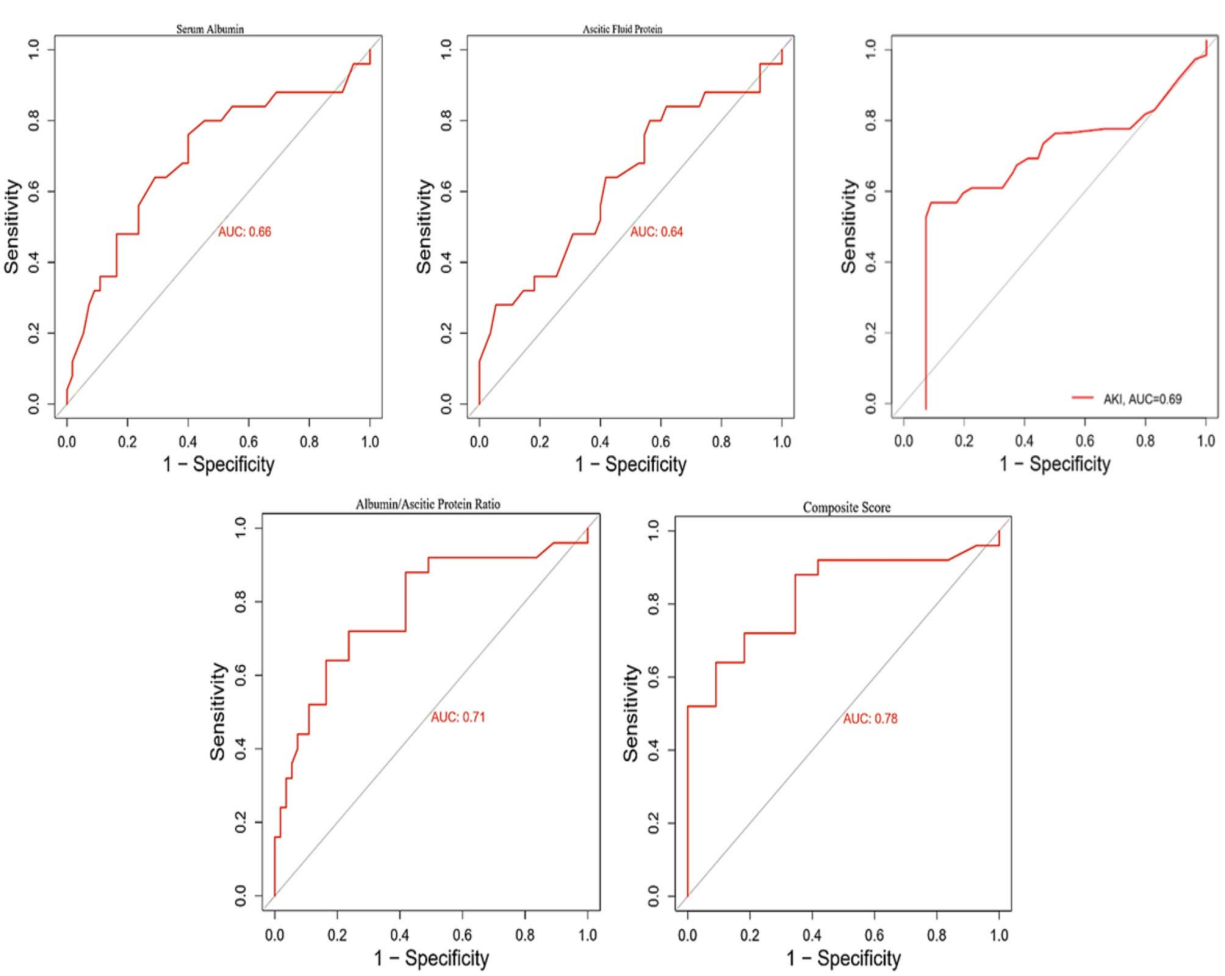
Receiver operating characteristic (ROC) curves for predictors of SBP recurrence. ROC analysis of key variables showed modest discrimination for serum albumin (AUC = 0.66), ascitic fluid protein (AUC = 0.64), and acute kidney injury (AKI, AUC = 0.69). The albumin/ascitic protein ratio demonstrated slightly higher discrimination (AUC = 0.71). The composite risk score, which integrates multiple predictors, achieved the highest discriminatory ability (AUC = 0.78), underscoring the benefit of combining clinical, biochemical, and inflammatory markers into a unified predictive tool

When assessing individual parameters, serum albumin at a cut-off of 2.5 g/dL demonstrated moderate predictive accuracy (sensitivity 68%, specificity 65%, AUC 0.66), while ascitic fluid protein at a cut-off of 1.0 g/dL showed slightly lower accuracy (sensitivity 62%, specificity 61%, AUC 0.64). The albumin/ascitic protein ratio provided better diagnostic performance (sensitivity 74%, specificity 72%, AUC 0.71). The presence of acute kidney injury was also a significant predictor, with reasonable sensitivity (70%) and specificity (68%), and an AUC of 0.69. Notably, the composite score (≥ 3) outperformed all individual variables, achieving the highest sensitivity (80%), specificity (75%), and AUC (0.78), indicating superior overall diagnostic accuracy and clinical utility (Table 5; Fig. 2).

**Table 5:** ROC curve analysis of predictive discriminator including combined score

Parameter	Cut-off	Sensitivity (%)	Specificity (%)	PPV (%)	NPV (%)	+LR	-LR	AUC
**Serum Albumin**	2.5 g/dL	68	65	60	70	1.94	0.49	0.66
**Ascitic Fluid Protein**	1.0 g/dL	62	61	58	65	1.59	0.62	0.64
**Albumin/Ascitic Protein Ratio**	2.5	74	72	68	77	2.64	0.36	0.71
**AKI**	Yes/No	70	68	60	75	2.19	0.44	0.69
**Composite Score**	≥ 3	80	75	73	82	3.20	0.27	0.78

### Predictive model Comparison–logistic regression (baseline supervised model) vs. machine learning models

**Fig. 3:**
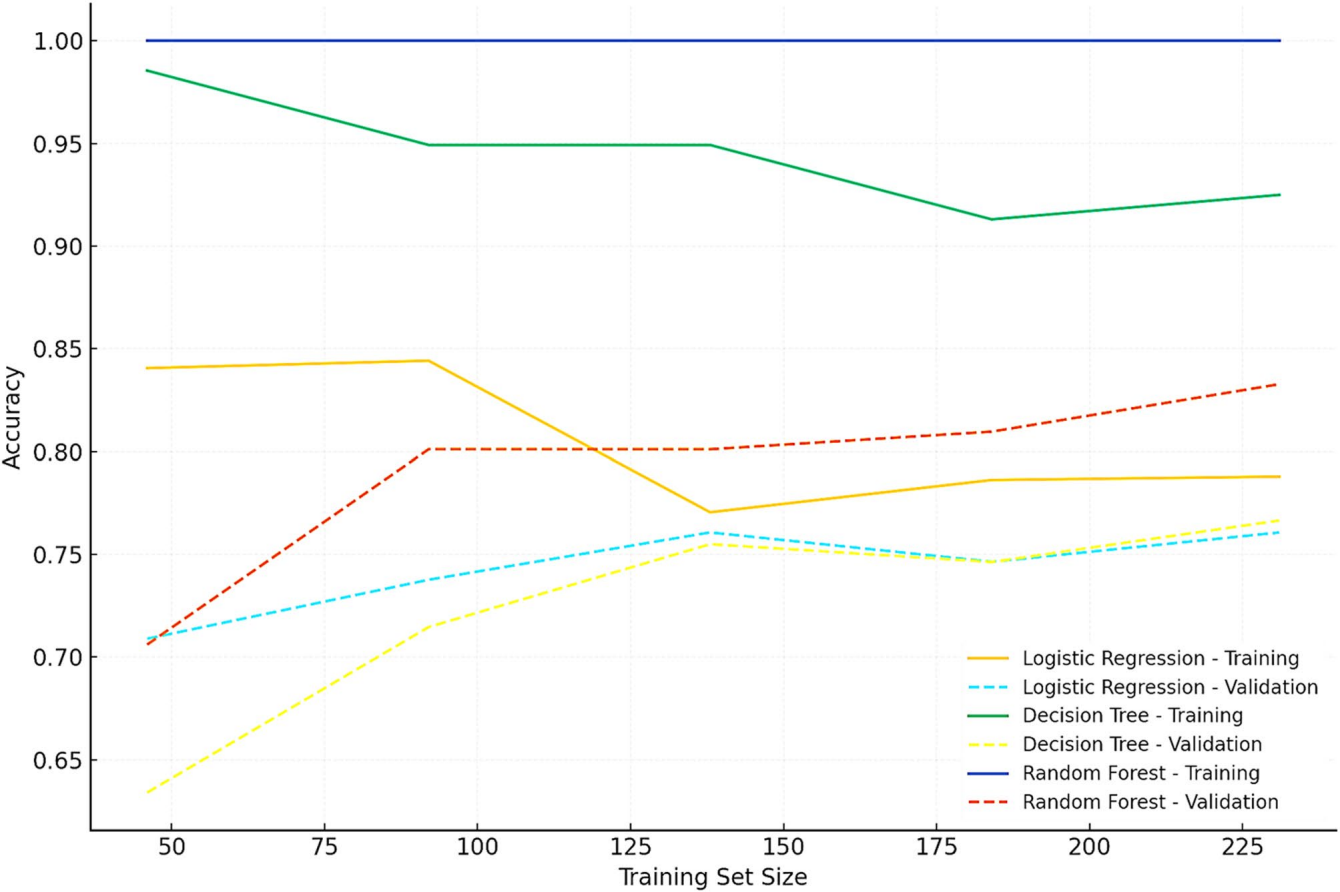
Learning curves for predictive models of SBP recurrence. Learning curves demonstrate training and validation accuracy as a function of training set size. Logistic regression improved gradually but plateaued at a lower performance. Decision tree models showed high training accuracy but lower validation accuracy, consistent with overfitting. Random forest models achieved the best generalization, with training and validation curves closely aligned, supporting its superior predictive performance

Table 6 compares the performance of logistic regression, decision tree, and random forest models in predicting SBP recurrence. Logistic regression (baseline supervised model) achieved an accuracy of 70%, sensitivity of 65%, specificity of 68%, and an AUC of 0.70. The decision tree demonstrated moderate performance, with an accuracy 75%, a sensitivity 72%, a specificity of 71%, and an AUC of 0.67. The random forest model achieved the highest performance, with an accuracy 78%, a sensitivity 77%, a specificity of 76%, and an AUC of 0.78. Statistical comparisons confirmed these findings. DeLong’s test revealed that the random forest AUC was significantly higher than logistic regression (*p* = 0.01) and decision tree (*p* = 0.004). McNemar’s test further demonstrated that random forest achieved significantly higher sensitivity and overall accuracy compared with both logistic regression and decision tree (*p* < 0.05). Learning curve analysis (Fig. 3) supported these results. Logistic regression improved gradually with increasing sample size but plateaued below the random forest performance. The decision tree achieved high training accuracy but lower validation accuracy, indicating overfitting. In contrast, random forest demonstrated consistently high training and validation accuracy, reflecting strong generalization and stable performance as the sample size increased.

**Table 6:** Predictive performance of supervised machine learning models (logistic regression, decision tree, and random forest) for SBP recurrence

Model	Accuracy (%)	Sensitivity (%)	Specificity (%)	AUC	Comparison vs. Random Forest (*p*)
**Logistic Regression**	70	65	68	0.70	AUC: 0.01 (DeLong); Sensitivity & Accuracy: <0.05 (McNemar)
**Decision Tree**	75	72	71	0.67	AUC: 0.004 (DeLong); Sensitivity & Accuracy: <0.05 (McNemar)
**Random Forest**	78	77	76	0.78	Reference model

Logistic regression served as the baseline supervised model. Random forest consistently outperformed logistic regression and decision tree in AUC (DeLong’s test) and classification accuracy/sensitivity (McNemar’s test). *p* < 0.05 was considered statistically significant after Bonferroni correction

## Discussion

In this study, the recurrence rate of SBP was 23.9%. Significant factors predicting recurrent SBP included acute kidney injury, elevated CRP levels, low serum albumin levels, high serum bilirubin levels, reduced β-blocker use, increased proton-pump inhibitor use, and a higher MELD Score (odds ratio > 1 for all). Additionally, the albumin/ascitic fluid protein ratio showed an inverse association with recurrent SBP (OR = 0.80).

In this cohort, renal dysfunction, systemic inflammation, and impaired nutritional status emerged as the most significant predictors of recurrent SBP.Renal injury is a frequent complication of SBP and a key predictor of poor outcomes in patients with cirrhosis. Research has shown that renal dysfunction is the primary prognostic factor, as indicated by the Model for End-Stage Liver Disease (MELD) score [21–23]. Serum creatinine is usually used for diagnosing acute kidney injury according to KDIGO and ICA-ADQI definitions [19], and higher creatinine levels have been linked to both increased SBP risk and worse outcomes [24, 25]. This indicates that kidney dysfunction is not merely a secondary manifestation but an active driver of adverse prognosis in SBP [26–30]. In our study, patients with recurrent SBP exhibited significantly higher creatinine levels, and AKI was identified as the strongest independent predictor of recurrence (OR = 3.1), reinforcing the central role of renal compromise in shaping outcomes [23]. Beyond renal dysfunction, systemic inflammation also emerged as an important factor. Elevated levels of C-reactive protein (CRP) were found to be significantly linked to recurrence (OR = 1.05), indicating that even slight increases can increase the risk. CRP, an acute-phase reactant, has previously been identified as a predictor of SBP recurrence [14, 26]., reflecting both the inflammatory burden and, in some cases, incomplete infection control [30]. Our findings confirm its utility as a biomarker of recurrence risk and align with prior reports linking elevated CRP with worse outcomes in cirrhotic patients [14, 26, 27, 31].

The nutritional and immunological status, as indicated by serum albumin levels, was found to be a significant predictor of recurrent SBP. Patients with recurrent SBP had notably lower albumin levels, and for each 1-unit decrease, there was a 10% higher risk of recurrence (OR = 1.1, *p* < 0.001). Hypoalbuminemia increases the risk of SBP by causing intestinal mucosal edema, bacterial translocation, and compromised immune defenses. These results align with previous research that has identified low albumin as a prognostic factor in SBP [6, 34].

What distinguishes this cohort is the combined predictive value of these three factors—renal impairment, systemic inflammation, and hypoalbuminemia—which together outline a high-risk profile for recurrence. While each factor has been individually associated with poor outcomes in prior studies, our results highlight their convergence in predicting SBP recurrence. This suggests that integrated monitoring of kidney function, inflammatory response, and nutritional status could improve risk stratification and guide targeted interventions to reduce recurrence in cirrhotic patients with SBP.

Our research revealed that patients with recurrent SBP had significantly higher serum bilirubin levels compared to those without recurrent SBP (*p* = 0.02). Bilirubin was also identified as a significant predictor of recurrent SBP, with an odds ratio of 1.4 and *p* = 0.02. These results indicate a strong association between elevated serum bilirubin levels and recurrent SBP, which aligns with findings from other studies [28, 29].

In our study, the group with recurrent SBP had a significantly lower rate of β-blockers usage and a higher rate of proton pump inhibitor (PPIs) usage compared to the non-recurrent SBP group. Both βB and PPIs were identified as predictors of recurrent SBP, with odds ratios of 2.5 and 1.6, respectively. The role of β-blockers has evolved over the years. In patients with cirrhosis, non-selective β-blockers (NSBBs) have shown promise in reducing the risk of complications such as ascites, refractory ascites, variceal hemorrhage, hepatorenal syndrome, and SBP [22, 30]. However, a study by Serste et al. has raised alarms about the safety of NSBBs in patients with cirrhosis and refractory ascites [31]. This study suggests potential harm, including increased mortality in patients with cirrhosis and refractory ascites. This finding has sparked a debate within the medical community regarding the use of NSBBs in patients with cirrhosis and ascites, with some recommending caution or avoidance of their use. In our study, we observed that patients with recurrent SBP had a significantly lower use of β-blockers compared to those with non-recurrent SBP (40 patients, 48%) versus 186 patients, 70%). β-blockers usage was also identified as a predictor of recurrent SBP. An OR of 2.5 indicates that patients not taking β-blockers have 2.5 times higher odds of recurrent SBP compared to those who are taking β-blockers. This suggests that β-blockers may have a protective effect in preventing the recurrence of SBP in this patient population, and their use is associated with a decreased rate of recurrence. In a study by Senzolo et al. [32], a potential beneficial effect of non-selective beta-blocker treatment on the development of SBP was suggested, based on a positive meta-analysis.

On the other hand, Mandorfer et al. [30] discovered that NSBBs are linked to adverse outcomes in patients with SBP, including reduced transplant-free survival, longer hospital stays, and higher rates of AKI. They also found that NSBBs increase the risk of complications and mortality in patients with cirrhosis and SBP [10]. These conflicting findings highlight the need for further research to determine the role of β-blockers in the management of SBP in patients with ascites.

The mechanism through which PPIs may elevate the risk of SBP in cirrhotic patients is not well understood. PPI use has been suggested to potentially increase this risk by promoting the growth of intestinal bacteria due to the suppression of gastric acid secretion [33]. Proton pump inhibitors can contribute to small intestinal bacterial overgrowth and bacterial translocation across the intestinal epithelial barrier to the lymph nodes by affecting gastrointestinal motility and increasing intestinal permeability [34]. In this study, proton-pump inhibitors were significantly more commonly used in recurrent SBP cases than in non-recurrent cases (56 patients, 68% versus 150 patients, 57%). Furthermore, PPI usage is a significant predictor of recurrent SBP with an odds ratio of 1.6. These findings suggest that PPI use might contribute to the recurrence of SBP in cirrhotic patients, possibly by altering the gut microbiome or increasing bacterial translocation. In line with our results, a large meta-analysis found that PPI use was associated with an increased risk of SBP in patients with cirrhosis and ascites [35]. A large study including 377,420 cirrhotic patients in the USA found that PPI use and hepatic encephalopathy posed the highest risk for the development of SBP. Cirrhotic patients using proton pump inhibitors are at a twofold risk of spontaneous bacterial peritonitis, independent of gastrointestinal bleeding [36]. This finding highlights the need for cautious use of PPIs in cirrhotic patients with ascites, especially considering the potential for an increased risk of SBP.

In our study, increasing MELD score was found to be a strong predictor of recurrent SBP, with an odds ratio of 2.9, indicating a significantly higher likelihood of repeat SBP episodes as the MELD score increases. This finding is consistent with a previous study that highlighted the MELD score as an important predictor for SBP [37–40].

In this study, a higher albumin/ascitic fluid protein ratio is associated with a reduced likelihood of SBP recurring (OR 0.80). This suggests that a higher albumin/ascitic fluid protein ratio is linked to a lower risk of recurrent SBP, indicating that the likelihood of recurrence decreases as the protein ratio increases. A lower albumin/ascitic fluid protein ratio may indicate a reduced ability of the ascitic fluid to resist bacterial infection. Ascitic proteins are essential for defending against bacterial infections, especially SBP in cirrhotic patients. Complement 3 is a key protein that directly fights bacteria in the ascitic fluid. In advanced cirrhosis, the liver produces less C3 and other proteins, weakening this defense and raising the infection risk. Additionally, albumin’s play an important role in opsonization, making bacteria more susceptible to phagocytosis. Also, hypoalbuminemia and increasing serum bilirubin are associated with decreased serum complement levels, which could lead to increased susceptibility to infections [10, 28, 29, 41].

In our study, a scoring system is proposed to predict the recurrence of SBP. Points are assigned based on specific thresholds for seven parameters: presence of AKI, CRP (> 50 mg/L), serum albumin < 2.5 g/dL, ascetic fluid protein < 1.0 g/dL, and an albumin/ascitic protein ratio < 2.5, each contributing 1–2 points. Additionally, a MELD score of ≥ 15, the presence of diabetes, and multidrug-resistant organisms each contribute 1–2 points, resulting in a potential total of 9 points. This score aids in stratifying patients according to their risk of recurrence by considering multiple inflammatory, biochemical, and clinical markers for a comprehensive risk assessment.

In our scoring system, integrated parameters were selected based on their prognostic value for recurrence, as supported by prior studies. For instance, AKI [39, 42, 43], CRP [18, 44, 45], and hypoalbuminemia [46, 47] have been consistently associated with poor outcomes in liver-related complications and infections, including SBP, due to reduced immune defense [10, 41]. Additionally, the MELD score is a strong predictor of SBP [37–39]. Furthermore, diabetes and multidrug-resistant organisms increase the risk of SBP in cirrhotic patients. Diabetes is an independent risk factor [48]. Antibiotic use and prolonged hospital stays also increase the risk of SBP and lead to poorer outcomes [48, 49], possibly due to metabolic dysfunction and antimicrobial resistance. Combining these markers as a composite score strengthens its predictive power by addressing multiple facets of disease pathology, including liver function, immune competence, and metabolic health. This system could be a practical tool for clinicians in triaging patients at higher risk of recurrence.

Our study uses a risk stratification model to classify patients based on their scores. Patients with scores of 0–3 are considered low risk, with a 15% recurrence rate. Those scoring 3–6 are categorized as moderate risk, with a 45% recurrence rate. Patients scoring 6–10 are classified as high risk, with an 80% recurrence rate. This model shows that higher scores are associated with a higher likelihood of recurrence, aiding in patient care and monitoring. The stratification method effectively identifies patients at the highest risk of recurrence, with an 80% recurrence rate in the high-risk group (scores 6–10). These findings are comparable to studies on similar risk stratification models, showing that higher scores correlate well with clinical deterioration and recurrence risk in liver disease and SBP [50]. This classification provides a clear clinical pathway and intervention; high-risk patients may benefit from more frequent follow-up or preemptive treatments to manage potential recurrence. The score’s application in clinical practice could significantly reduce adverse outcomes by enabling early intervention for high-risk patients.

In this study, the ROC curve analysis of predictive discriminators (serum albumin, ascitic fluid protein, albumin/ascitic protein ratio, and AKI) showed that the composite score, with an AUC of 0.78, is a highly effective predictor of SBP recurrence. The composite score’s sensitivity (80%) and specificity (75%) outperform individual biomarkers. These findings emphasize the superior predictive ability of combined scoring metrics over individual markers. These findings are aligned with earlier studies, where composite scoring systems have consistently shown higher predictive accuracy than single markers in conditions with complex pathophysiologies, such as SBP, especially in the setting of statistical efficiency and reducing sample size requirements [51].

In this study, a comparison between logistic regression (supervised machine learning (ML) baseline), decision tree, and random forest algorithms was conducted to assess their predictive capabilities. The results showed that the random forest model outperformed the other models, achieving the highest performance metrics with 78% accuracy, 77% sensitivity, and 76% specificity (AUC 0.78). In comparison, the decision tree model had an accuracy of 75%, sensitivity of 72%, specificity of 71%, and an AUC of 0.75, while logistic regression achieved an accuracy of 70%, sensitivity of 65%, specificity of 68%, and an AUC of 0.70. The random forest ML model demonstrated superior performance across all evaluated metrics compared to logistic regression. It showed an 8% higher accuracy, 7% higher sensitivity, 8% higher specificity, and a 0.08 increase in AUC, indicating its better overall predictive capability in this specific comparison. Our findings highlight the complementary value of predictive modeling in assessing SBP recurrence risk. Logistic regression, while clinically interpretable, achieved only moderate discrimination (AUC 0.70) and plateaued in learning curve analysis, reflecting limited capacity to capture nonlinear interactions. The decision tree offered slightly higher accuracy but showed overfitting, as evidenced by the divergence between training and validation performance on the learning curve.

In contrast, the random forest ensemble consistently outperformed both models, achieving the highest AUC (0.78) and balanced accuracy, sensitivity, and specificity. Learning curve analysis confirmed that random forest generalized well to unseen data, with closely aligned training and validation accuracy across sample sizes. This advantage is attributable to its bagging and feature randomness strategy, which reduces variance and improves stability, compared to single decision trees. Taken together, these results underscore that while logistic regression remains valuable for interpretability, ensemble-based approaches such as random forest offer superior predictive reliability in complex clinical settings like SBP recurrence. The inclusion of learning curves provides further evidence that random forest achieves robust generalization, thereby justifying its clinical application as a supportive decision-making tool. These findings align with contemporary studies that highlight ML advantages in handling complex, multidimensional datasets that are standard in medical research. We appreciate the positive feedback. Random forest attained superior predictive performance v logistic regression and decision trees due to its ensemble bagging strategy, which decreases variance, captures nonlinear interactions, and enhances generalizability. Although logistic regression remains clinically intuitive, random forest provides complementary value. Still, interpretability can be enhanced through variable importance rankings and SHAP (Shapley Additive Explanations), bridging the gap between predictive power and clinical insight [52, 53]. The decision tree and random forest models excel in predictive accuracy and could be particularly beneficial in real-time clinical decision-making. Incorporating these models could optimize risk prediction and improve patient outcomes, illustrating a shift toward data-driven healthcare solutions in managing chronic and recurrent conditions like SBP.

Despite the thorough design and strong predictive modeling, this study has limitations to consider. It was conducted at two centers in a single country, short follow-up, lacking external validation, which may limit the generalizability of the findings to other populations or healthcare settings with different microbiological profiles or clinical practices. The study used creatinine-based definitions for AKI diagnosis, which may not fully detect early kidney injury in cirrhotic patients due to altered muscle mass and fluid status. While machine learning models improved predictive accuracy, their performance could vary with different datasets, highlighting the need for external validation. These limitations emphasize the importance of multicenter prospective validation studies with extended follow-up and biomarker refinement to validate and optimize the proposed predictive scoring system.

## Conclusion

Our study emphasizes the importance of a comprehensive, multi-parametric approach to predict SBP recurrence. By incorporating acute kidney injury, clinical and biochemical biomarkers, and supervised machine learning models, especially random forest, we establish a robust framework for risk stratification. However, validation in larger, multicenter cohorts is necessary.

## Data Availability

The data used in this study is available upon a reasonable request to the corresponding author and after permission from all participating services.

## References

[1] Aithal GP, Palaniyappan N, China L, Härmälä S, Macken L, Ryan JM, et al. Guidelines on the management of ascites in cirrhosis. Gut. 2021;70(1):9–29. 33067334 10.1136/gutjnl-2020-321790 PMC7788190

[2] De Franchis R, Bosch J, Garcia-Tsao G, Reiberger T, Ripoll C, Abraldes JG, et al. Baveno VII–renewing consensus in portal hypertension. J Hepatol. 2022;76(4):959–74. 35120736 10.1016/j.jhep.2021.12.022 PMC11090185

[3] Niu C, Zhang J, Himal K, Zhu K, Zachary T, Verghese B, Jadhav N, Okolo PI, Daglilar E, Kouides P. Impact of anticoagulation therapy on outcomes in patients with cirrhosis and portal vein thrombosis: A large-scale retrospective cohort study. Thrombosis Research. 2024; 241:109103. 10.1016/j.thromres.2024.109103 39067278

[4] Niu C, Zhang J, Daniel-Jose ID, Zachary T, Abdullah O, Shah P,Basant E, Maity D, Abdullah FA, Jadhav N, Okolo PI, Ebubekir D. Anticoagulationoutcomes in cirrhotic patients with portal vein thrombosis: a tertiary center study. PostgradMed J. 2025 Oct 18;101(1201):1166-1172. 10.1093/postmj/qgaf062 40298249

[5] Evans LT, Kim RW, Poterucha JJ, Kamath PS. Spontaneous bacterial peritonitis in asymptomatic outpatients with cirrhotic ascites. Hepatology. 2003;37(4):897–901. 12668984 10.1053/jhep.2003.50119

[6] Oliveira AM, Branco JC, Barosa R, Rodrigues JA, Ramos L, Martins A, et al. Clinical and microbiological characteristics associated with mortality in spontaneous bacterial peritonitis: a multicenter cohort study. Eur J Gastroenterol Hepatol. 2016;28(10):1216–22. 27391170 10.1097/MEG.0000000000000700

[7] Mousa N, Salah M, Elbaz S, Elmetwalli A, Elhammady A, Abdelkader E, et al. Neutrophil percentage-to-albumin ratio is a new diagnostic marker for spontaneous bacterial peritonitis: a prospective multicenter study. Gut Pathog. 2024;16(1):18. 38561807 10.1186/s13099-024-00610-2 PMC10985869

[8] El Shabrawi A, Abdelaziz M, Mousa N. Infections in cirrhotic patients. Med J Viral Hepat. 2019;4(1):5–14.

[9] Christou L, Pappas G, Falagas ME. Bacterial infection-related morbidity and mortality in cirrhosis. Am J Gastroenterol. 2007;102(7):1510–7. 17509025 10.1111/j.1572-0241.2007.01286.x

[10] Huang CH, Lin CY, Sheen IS, Chen WT, Lin TN, Ho YP, et al. Recurrence of spontaneous bacterial peritonitis in cirrhotic patients non-prophylactically treated with norfloxacin: serum albumin as an easy but reliable predictive factor. Liver Int. 2011;31(2):184–91. 21143367 10.1111/j.1478-3231.2010.02377.x

[11] França AVC, De Souza JB, Silva CM, Soares EC. Long-term prognosis of cirrhosis after spontaneous bacterial peritonitis treated with ceftriaxone. J Clin Gastroenterol. 2001;33(4):295–8. 11588542 10.1097/00004836-200110000-00007

[12] Amr S, Ahmed A-R, Hoda E. Spontaneous bacterial peritonitis: an overview. Med J Viral Hepat. 2018;3(1):13–7.

[13] Termsinsuk P, Auesomwang C. Factors that predict recurrent spontaneous bacterial peritonitis in cirrhotic patients. Int J Clin Pract. 2020;74(3):e13457. 31799716 10.1111/ijcp.13457

[14] Titó L, Rimola A, Ginès P, Llach J, Arroyo V, Rodés J. Recurrence of spontaneous bacterial peritonitis in cirrhosis: frequency and predictive factors. Hepatology. 1988;8(1):27–31. 3257456 10.1002/hep.1840080107

[15] Abdel-Razik A, Mousa N, Abdel-Aziz M, Elsherbiny W, Zakaria S, Shabana W, et al. Mansoura simple scoring system for prediction of spontaneous bacterial peritonitis: lesson learnt. Eur J Gastroenterol Hepatol. 2019;31(8):1017–24. 30694910 10.1097/MEG.0000000000001364

[16] Thalheimer U, Triantos CK, Samonakis DN, Patch D, Burroughs AK. Infection, coagulation, and variceal bleeding in cirrhosis. Gut. 2005;54(4):556–63. 15753544 10.1136/gut.2004.048181 PMC1774431

[17] Dam G, Vilstrup H, Watson H, Jepsen P. Proton pump inhibitors as a risk factor for hepatic encephalopathy and spontaneous bacterial peritonitis in patients with cirrhosis with ascites. Hepatology. 2016;64(4):1265–72. 27474889 10.1002/hep.28737

[18] Mousa N, Besheer T, Abdel-Razik A, Hamed M, Deiab AG, Sheta T, et al. Can combined blood neutrophil to lymphocyte ratio and C-reactive protein be used for diagnosis of spontaneous bacterial peritonitis? Br J Biomed Sci. 2018;75(2):71–5. 29452544 10.1080/09674845.2017.1396706

[19] Shi K-Q, Fan Y-C, Ying L, Lin X-F, Song M, Li L-F, et al. Risk stratification of spontaneous bacterial peritonitis in cirrhosis with ascites based on classification and regression tree analysis. Mol Biol Rep. 2012;39(5):6161–9. 22205541 10.1007/s11033-011-1432-8

[20] Cao NH, Ho PT, Bui HH, Vo TD. Non-invasive methods for the prediction of spontaneous bacterial peritonitis in patients with cirrhosis. Gastroenterol Insights. 2023;14(2):170–7.

[21] Ono S, Goto T. Introduction to supervised machine learning in clinical epidemiology. Annals of Clinical Epidemiology. 2022;4(3):63–71. 38504945 10.37737/ace.22009 PMC10760492

[22] Angeli P, Bernardi M, Villanueva C, Francoz C, Mookerjee RP, Trebicka J, et al. EASL clinical practice guidelines for the management of patients with decompensated cirrhosis. J Hepatol. 2018;69(2):406–60. 29653741 10.1016/j.jhep.2018.03.024

[23] Runyon BA. Introduction to the revised American association for the study of liver diseases practice guideline management of adult patients with ascites due to cirrhosis 2012. Hepatology. 2013;57(4):1651–3. 23463403 10.1002/hep.26359

[24] Nadim MK, Kellum JA, Forni L, Francoz C, Asrani SK, Ostermann M, et al. Acute kidney injury in patients with cirrhosis: acute disease quality initiative (ADQI) and international club of ascites (ICA) joint multidisciplinary consensus meeting. J Hepatol. 2024;81(1):163–83. 38527522 10.1016/j.jhep.2024.03.031 PMC11193657

[25] Gupta R, Misra SP, Dwivedi M, Misra V, Kumar S, Gupta SC. Diagnosing ascites: value of ascitic fluid total protein, albumin, cholesterol, their ratios, serum-ascites albumin and cholesterol gradient. J Gastroenterol Hepatol. 1995;10(3):295–9. 7548806 10.1111/j.1440-1746.1995.tb01096.x

[26] Chung C, Iwakiri Y. The lymphatic vascular system in liver diseases: its role in ascites formation. Clin Mol Hepatol. 2013;19(2):99. 23837133 10.3350/cmh.2013.19.2.99 PMC3701854

[27] Chen SM, Lo GH, Lai KH, Cheng HH, Cheng JS, Shen MT, et al. Serum and ascitic concentration of C3, C4 and protein in cirrhotic patients with spontaneous bacterial peritonitis. Zhonghua Yi Xue Za zhi = Chinese medical Journal; free China ed. 1994;54(2):87–92. 7954051

[28] Moalla M, Elleuch N, Dahmani W, Hammami A, Ameur WB, Slama AB, et al. Predictive factors of recurrence in spontaneous bacterial peritonitis in Tunisian patients with cirrhosis. Future Sci OA. 2023;9(5):FSO857. 37180608 10.2144/fsoa-2023-0016 PMC10167715

[29] Jamil S, Ahmed S, Memon A, Masood S, Shah SH, Hamid SS, Jafri W. Factors predicting the recurrence of spontaneous bacterial peritonitis in patients with cirrhosis. JCPSP: J Coll Physicians Surgeons–Pakistan. 2011;21(7):407. 21777528

[30] Mandorfer M, Bota S, Schwabl P, Bucsics T, Pfisterer N, Kruzik M, Hagmann M, Blacky A, Ferlitsch A, Sieghart W. Nonselective β blockers increase risk for hepatorenal syndrome and death in patients with cirrhosis and spontaneous bacterial peritonitis. Gastroenterology. 2014;146(7):1680–90. 24631577 10.1053/j.gastro.2014.03.005

[31] Sersté T, Melot C, Francoz C, Durand F, Rautou PE, Valla D, et al. Deleterious effects of beta-blockers on survival in patients with cirrhosis and refractory ascites. Hepatology. 2010;52(3):1017–22. 20583214 10.1002/hep.23775

[32] Senzolo M, Cholongitas E, Burra P, Leandro G, Thalheimer U, Patch D, et al. β-blockers protect against spontaneous bacterial peritonitis in cirrhotic patients: a meta‐analysis. Liver Int. 2009;29(8):1189–93. 19508620 10.1111/j.1478-3231.2009.02038.x

[33] de Vos M, De Vroey B, Garcia BG, Roy C, Kidd F, Henrion J, et al. Role of proton pump inhibitors in the occurrence and the prognosis of spontaneous bacterial peritonitis in cirrhotic patients with ascites. Liver Int. 2013;33(9):1316–23. 23730823 10.1111/liv.12210

[34] van Vlerken LG, Huisman EJ, van Hoek B, Renooij W, de Rooij FWM, Siersema PD, et al. Bacterial infections in cirrhosis: role of proton pump inhibitors and intestinal permeability. Eur J Clin Invest. 2012;42(7):760–7. 22288900 10.1111/j.1365-2362.2011.02643.x

[35] Alhumaid S, Al Mutair A, Al Alawi Z, Zaidi ARZ, Rabaan AA, Elhazmi A, et al. Proton pump inhibitors use and risk of developing spontaneous bacterial peritonitis in cirrhotic patients: a systematic review and meta-analysis. Gut Pathog. 2021;13(1):17. 33741033 10.1186/s13099-021-00414-8 PMC7977161

[36] Boustany A, Rahhal R, Onwuzo S, Almomani A, Boustany T, Kumar P, Asaad I. Cirrhotic patients on proton pump inhibitors are at a twofold risk of spontaneous bacterial peritonitis independently of Gastrointestinal bleeding: a population-based retrospective study. Annals Gastroenterol. 2023;36(3):327. 10.20524/aog.2023.0794 PMC10152803 37144010

[37] Follo A, Llovet JM, Navasa M, Planas R, Forns X, Francitorra A, et al. Renal impairment after spontaneous bacterial peritonitis in cirrhosis: incidence, clinical course, predictive factors and prognosis. Hepatology. 1994;20(6):1495–501. 7982650 10.1002/hep.1840200619

[38] Tandon P, Garcia–Tsao G. Renal dysfunction is the most important independent predictor of mortality in cirrhotic patients with spontaneous bacterial peritonitis. Clin Gastroenterol Hepatol. 2011;9(3):260–5. 21145427 10.1016/j.cgh.2010.11.038 PMC3713475

[39] Siew ED, Parr SK, Abdel-Kader K, Eden SK, Peterson JF, Bansal N, et al. Predictors of recurrent AKI. J Am Soc Nephrol. 2016;27(4):1190–200. 26264853 10.1681/ASN.2014121218 PMC4814177

[40] Obstein KL, Campbell MS, Reddy KR, Yang Y-X. Association between model for end-stage liver disease and spontaneous bacterial peritonitis. Am J Gastroenterol. 2007;102(12):2732–6. 17714556 10.1111/j.1572-0241.2007.01485.x

[41] Hung T-H, Ko P-H, Wang C-Y, Tsai C-C, Lee H-F. Effect of hypoalbuminemia on mortality in cirrhotic patients with spontaneous bacterial peritonitis. Tzu Chi Med J. 2024;36(1):92–7. 38406576 10.4103/tcmj.tcmj_211_23 PMC10887335

[42] El-Gamal S, El-Menshawy HHE-B, Abbas NF, El-Metwally O. Acute kidney injury network criteria as a prognostic factor in cirrhotic patients with spontaneous bacterial peritonitis. Egypt J Intern Med. 2018;30(4):264–70.

[43] Nuthalapati A, Schluterman N, Khanna A, Greenberg D, Thuluvath PJ. Impact of acute kidney injury on mortality of patients hospitalized for complications of cirrhosis. J Clin Exp Hepatol. 2017;7(4):290–9. 29234192 10.1016/j.jceh.2017.05.004 PMC5720141

[44] Elmetwalli A, Hassan J, Ismail N, Rizk R, Abdelaziz M, Mohamed S, Salama H, Abdelkader E. C-reactive protein to albumin ratio: A new score for predicting the recurrence of spontaneous bacterial peritonitis in cirrhotic patients with Ascites. Med J Viral Hepat. 2024;8(2):6–8.

[45] Mousa N, Zakaria S, Maksoud MAEL, Shabana W, Effat N, Awad M, Abd Elmohsen Eldesoky TS, El-Wakeel N, Moemen D, Mikhail N. The predictive factors for recurrence of spontaneous bacterial peritonitis. Med J Viral Hepat. 2018;2(2):25–9.

[46] Schnabl B, Brenner DA. Interactions between the intestinal microbiome and liver diseases. Gastroenterology. 2014;146(6):1513–24. 24440671 10.1053/j.gastro.2014.01.020 PMC3996054

[47] Skinner C, Thompson AJ, Thursz MR, Marchesi JR, Vergis N. Intestinal permeability and bacterial translocation in patients with liver disease, focusing on alcoholic aetiology: methods of assessment and therapeutic intervention. Ther Adv Gastroenterol. 2020;13:1756284820942616. 10.1177/1756284820942616 PMC7580143 33149761

[48] Alhajaji R, Samkari MM, Althobaiti MA, Al-Ahmadi BR, Bugis AM, Bugis AM, et al. Diabetes mellitus and the risk of spontaneous bacterial peritonitis in patients with liver cirrhosis: a systematic review and meta-analysis. Ann Saudi Med. 2024;44(4):272–87. 39127903 10.5144/0256-4947.2024.272 PMC11316951

[49] de Mattos AA, Costabeber AM, Lionço LC, Tovo CV. Multi-resistant bacteria in spontaneous bacterial peritonitis: a new step in management? World J Gastroenterol. 2014;20(39):14079. 25339797 10.3748/wjg.v20.i39.14079 PMC4202339

[50] Huynh NC, Vo TD. Validation of a new simple scoring system to predict spontaneous bacterial peritonitis in patients with cirrhosis and ascites. BMC Gastroenterol. 2023;23(1):272. 37559036 10.1186/s12876-023-02919-9 PMC10411006

[51] Dash K, Goodacre S, Sutton L. Composite outcomes in clinical prediction modeling: are we trying to predict apples and oranges? Ann Emerg Med. 2022;80(1):12–9. 35339284 10.1016/j.annemergmed.2022.01.046

[52] Weissler EH, Naumann T, Andersson T, Ranganath R, Elemento O, Luo Y, et al. The role of machine learning in clinical research: transforming the future of evidence generation. Trials. 2021;22(1):537. 34399832 10.1186/s13063-021-05489-x PMC8365941

[53] Couronné R, Probst P, Boulesteix A-L. Random forest versus logistic regression: a large-scale benchmark experiment. BMC Bioinformatics. 2018;19(1):270. 30016950 10.1186/s12859-018-2264-5 PMC6050737

